# 45,X product of conception after preimplantation genetic diagnosis and euploid embryo transfer: evidence of a spontaneous conception confirmed by DNA fingerprinting

**DOI:** 10.1186/s12958-016-0190-9

**Published:** 2016-09-06

**Authors:** Daniela Bettio, Antonio Capalbo, Elena Albani, Laura Rienzi, Valentina Achille, Anna Venci, Filippo Maria Ubaldi, Paolo Emanuele Levi Setti

**Affiliations:** 1 Cytogenetic and Medical Genetic Laboratory, Operative Unit of Clinical Investigations, Humanitas Clinical and Research Center, Via Manzoni 56, 20089 Rozzano, Milan Italy; 2 GENERA, Reproductive Medicine Centers, Rome, Italy; 3 GENETYX, Molecular Genetics Laboratory, Via Fermi 1, Marostica, Vicenza Italy; 4 Department of Gynecology, Division of Gynecology and Reproductive Medicine, Fertility Center, Humanitas Clinical and Research Center, Via Manzoni 56, Rozzano, Milan Italy

**Keywords:** POC, PGS, Blastocyst transfer, Cytogenetics, SNPs

## Abstract

**Background:**

Preimplantation genetic screening (PGS) provides an opportunity to eliminate a potential implantation failure due to aneuploidy in infertile couples. Some studies clearly show that twins following single embryo transfer (SET) can be the result of a concurrent natural conception and an incidence as high as 1 in 5 twins has been reported.

In our case PGS was performed on trophectoderm (TE) biopsies by quantitative polymerase chain reaction (qPCR). The product of conception (POC) was cytogenetically investigated after selection of the placental villi by means of the direct method. Molecular cytogenetic characterization of the POC was performed by fluorescence in situ hybridization (FISH) and array-comparative genomic hybridization (a-CGH) analyses. To investigate the possibility of a spontaneous conception, a panel of 40 single nucleotide polymorphisms (SNPs) was used to compare genetic similarity between the DNA of the POC and the DNA leftover of the TE biopsy.

**Findings:**

We describe a 36-year old infertile woman undergoing PGS who had a spontaneous abortion after a single euploid embryo transfer on a spontaneous cycle. The POC showed a 45,X karyotype confirmed by FISH and a-CGH. DNA fingerprinting demonstrated a genetic similarity of 75 % between the DNA of the POC and TE biopsy, consistent with a sibling status. All supernumerary euploid embryos were also tested showing a non-self relationship with the POC, excluding a mix-up event at the time of fetal embryo transfer.

**Conclusions:**

DNA fingerprinting of the transferred blastocyst and POC, confirmed the occurrence of a spontaneous conception. This case challenges the assumption that a pregnancy after assisted reproductive technology (ART) is always a result of ART, and strengthens the importance to avoid intercourses during PGS and natural transfer cycles. Moreover, cytogenetic analysis of the POCs is strongly recommended along with fingerprinting children born after PGS to see what the concordance is between the embryo transferred and the resultant child.

## Introduction

PGS is an early method to detect aneuploidies in preimplantation embryos. PGS can be offered to patients with an increased risk of having a higher percentage of chromosomally abnormal embryos, improving the reproductive outcome. By transferring only chromosomally normal embryos the miscarriage rates are expected to be lower, since about 60–70 % of miscarriages in the first trimester result from chromosomal abnormalities in the fetus [1].

Some studies clearly show that twins following SET can be the result of a concurrent natural conception [2, 3] and an incidence as high as 1 in 5 twins has been reported [4].

We describe a rare case of a 36-year-old infertile patient with recurrent implantation failures. PGS was performed and an euploid blastocyst transferred. However, a spontaneous abortion occurred after 13 weeks of gestation, ascertained to be 10 at ultrasound examination. Cytogenetic analysis revealed a 45,X karyotype. DNA fingerprinting analysis of POC and the euploid embryo transferred excluded a self-relationship confirming the occurrence of a spontaneous conception in concomitance with SET.

## Materials and methods

A 36-year old woman experiencing 6 years infertility with a diagnosis of severe endometriosis was referred to Humanitas Fertility Center. She had a miscarriage at 8 weeks after an intrauterine insemination (IUI), other 2 negative IUI cycles and 3 negative in vitro fertilization (IVF) cycles with the transfer of 11 cleavage stage embryos from fresh and frozen cycles performed in other centers. A normal uterine cavity was confirmed at hysteroscopy while a transvaginal ultrasound imaging showed a 18 mm small endometrial cyst of the right ovary and 12 mm recto-vaginal lesion. Despite a high follicle-stimulating hormone (FSH) level of 14 IU/L, ovarian reserve assessed to be normal with an anti-Müllerian hormone (AMH) level of 3.8 ng/ml and the existence of 10 antral follicles. Her partner sperm analysis showed 12 million total motile sperm with 2 % normal forms and 80 % vitality. After counseling and obtaining informed consent, the patient was enrolled for a first attempt intracytoplasmic sperm injection (ICSI) with PGS cycle. Controlled ovarian hyper-stimulation was started with 225 IU recombinant FSH (rFSH) in a gonadotropin-releasing hormone (GnRH) antagonist analog cycle. Recombinant human chorionic gonadotropin (rhCG) (250 mcg) was administered on day 13 of the induction cycle after a total rFSH dose of 3325 IU.

A transvaginal ultrasound guided under deep sedation oocyte retrieval was performed 36 h after the rhCG trigger and 9 metaphase II oocytes were retrieved. In 8 metaphase II oocytes ICSI was performed and placed in an incubator with an integrated time-lapse system, where the embryos were cultured individually in 25 μl microwells and monitored for 24 h. At 16–18 h post-ICSI, the 8 oocytes were assessed for the presence of 2 pronuclei and cultured until blastocyst stage. Three expanded blastocysts underwent biopsy of TE cells day 5 and 4 day 6 as previously described [5], 1 embryo degenerated after development to morula. Blastocyst quality was assessed immediately before TE biopsy [6], and blastocysts vitrified according to the protocol described by Kuwayama et al. [7]. Of the 7 biopsied and cryopreserved blastocysts, 4 were euploid. TE biopsies were analysed by qPCR based comprehensive chromosome diagnosis [8, 9]. This method has been extensively validated for chromosome copy number analysis of TE biopsies in preclinical [8, 9] and clinical studies [10, 11] and was reported to have a recognizable error rate of 0.2 % in clinical pregnancies [12].

The embryo transfer was scheduled on a spontaneous cycle. The patient was monitored from day 6 of the cycle by ultrasound and a urinary luteinizing hormone (LH) kit. On cycle day 15 an endometrial thickness of 12.2 mm with 2 follicles of 18 and 22 mm were observed with a positive LH determination. Seven days after LH surge a single euploid blastocyst (embryo number 3) was selected for transfer based on morphological score, warmed and cultured at 37 °C until transfer, performed under ultrasound guidance.

A positive hCG determination was performed 14 days after the embryo transfer (1765 IU) and a 6 weeks ultrasound showed an intrauterine gestation with an embryo with normal fetal heart rate.

A diagnosis of internal abortion was performed at 13 weeks with a 10 weeks embryo development and after the diagnosis the patients was submitted to aspiration and curettage with selection of villous materials immediately sent to our Cytogenetic Laboratory.

Placental villi were then further selected under the inverted microscope and cytogenetic analysis performed by direct preparation after 24 h incubation [13]. This is the method of choice in our laboratory because it avoids the growth of normal cells from possible endometrial contamination [1]. Parental karyotyping was performed according to standard procedures.

FISH on 1000 interphase nuclei using Vysis CEPX (DXZ1) Spectrum Green Probe (Abbott Molecular, Abbott Park, Illinois, US), specific for chromosome X centromere was conducted following the manufacturer’s recommendations.

Array-CGH study was performed on the DNA extracted from uncultured villi using the QIAamp Mini Kit (QIAGEN®, Hilden, Germany) and hybridized to the SurePrint G3 Cancer CGH + SNP 4 × 180 K microarray platform (Agilent Technologies, Santa Clara, CA, USA). Data were analysed with Agilent CytoGenomics 3.0.1.1 software.

Since the couple did not abstain from having sexual intercourses during the embryo transfer period, DNA fingerprinting analysis of excess embryonic and POC DNA was carried out to rule out the hypothesis of a spontaneous conception rather than a false PGS negative result. A set of 40 previously published SNPs with high heterozygosity and low allele frequency variation were utilized [14]. These SNPs were originally characterized against ~2070 individuals from 40 populations. The estimated probability that siblings will have identical genotypes is 0.5 raised to the power of the number of SNPs used, since all of these markers have alleles close to 50:50 in the population. This technique has been validated by Scott and colleagues [15] as the first seamless and inexpensive trophectoderm qPCR-based DNA fingerprinting technology allowing the unequivocal discrimination of sibling human embryos and foetuses. Briefly, for either the multiplex amplification PCR product residual from the TE biopsy or the purified genomic DNA of the POC, real-time PCR for allelic discrimination was performed in duplicate reactions for each of the individual 40 loci. Each reaction used the individual TaqMan SNP Genotyping Assays, TaqMan Genotyping Master Mix (Applied Biosystems Inc.), a 5 μl reaction volume, a 384-well plate, and a Quant Studio sequence detection system, as recommended by the supplier (Applied Biosystems Inc.). Informative SNP genotypes were compared between samples to determine the levels of similarity and standard deviations from the mean were calculated.

## Results and discussion

The couple’s karyotype was normal. Cytogenetic analysis on 20 Q-banded metaphases obtained from the POC showed a 45,X karyotype. This result was confirmed by FISH analysis showing one signal for CEPX in 94.9 % of the nuclei. In 5.1 % of nuclei 2 signals were present, probably due to a small maternal cell contamination that is difficult to avoid in a POC sample even after placenta villi selection. The 45,X karyotype observed in the metaphases obtained after 24 h of incubation excluded a mosaic karyotype in the embryo.

Array-CGH analysis performed on DNA from uncultured villi confirmed chromosome X monosomy.

Allelic discrimination analysis of 40 SNPs of the excess DNA from all the euploid blastocysts produced during the present IVF cycle, revealed that none of them had a genotype consistent with the POC. In particular, locus dropout (no amplification of either allele) was observed in only 4 assays of embryo number 3, with an overall call rate of 97.5 % (156/160) (Table 1). All the euploid embryos showed a similarity rate to POC below 80 %, clearly consistent with a sibling genotype and a non-self-relationship (Table 2). This analysis confirmed the hypothesis of a spontaneous conception in the course of the natural frozen embryo transfer cycle.

**Table 1:** Genotyping data of 40 SNPs in technical duplicates used to determine genetic similarity between the product of conception and euploid IVF embryos. 1 and 2 represents the two alleles for each SNP

Assay ID	Villi gDNA	Embryo 3	Embryo 6	Embryo 7
Assay01	11	11	22	11
Assay01	11	11	22	11
Assay02	22	unamplified	22	22
Assay02	22	unamplified	22	22
Assay03	11	11	11	11
Assay03	11	11	11	11
Assay04	11	11	11	12
Assay04	11	11	11	12
Assay05	12	22	12	12
Assay05	12	22	12	12
Assay06	22	22	12	22
Assay06	22	22	12	22
Assay07	12	11	11	12
Assay07	12	11	11	12
Assay08	22	22	22	22
Assay08	22	22	22	22
Assay09	12	unamplified	12	12
Assay09	12	unamplified	12	12
Assay10	12	11	11	11
Assay10	12	11	11	11
Assay11	11	12	12	11
Assay11	11	12	12	11
Assay12	12	12	12	12
Assay12	12	12	12	12
Assay13	11	11	11	11
Assay13	11	11	11	11
Assay14	22	11	22	12
Assay14	22	11	22	12
Assay15	22	22	12	12
Assay15	22	22	12	12
Assay16	12	12	11	22
Assay16	12	12	11	22
Assay17	12	12	11	12
Assay17	12	12	11	12
Assay18	12	12	12	12
Assay18	12	12	12	12
Assay19	12	12	22	12
Assay19	12	12	22	12
Assay20	11	11	11	11
Assay20	11	11	11	11
Assay21	12	12	22	12
Assay21	12	12	22	12
Assay22	22	22	12	22
Assay22	22	22	12	22
Assay23	11	12	12	12
Assay23	11	12	12	12
Assay24	22	22	12	12
Assay24	22	22	12	12
Assay25	12	12	12	12
Assay25	12	12	12	12
Assay26	22	22	12	22
Assay26	22	22	12	22
Assay27	22	12	22	12
Assay27	22	12	22	12
Assay28	12	12	12	12
Assay28	12	12	12	12
Assay29	11	12	11	11
Assay29	11	12	11	11
Assay30	12	12	12	12
Assay30	12	12	12	12
Assay31	12	12	22	12
Assay31	12	12	22	12
Assay32	22	22	22	22
Assay32	22	22	22	22
Assay33	11	11	12	11
Assay33	11	11	12	11
Assay34	22	22	22	12
Assay34	22	22	22	12
Assay35	22	22	22	22
Assay35	22	22	22	22
Assay36	11	11	11	11
Assay36	11	11	11	11
Assay37	12	11	11	11
Assay37	12	11	11	11
Assay38	12	12	12	12
Assay38	12	12	12	12
Assay39	11	12	11	11
Assay39	11	12	11	11
Assay40	11	11	12	11
Assay40	11	11	12	11

**Table 2:** Genetic similarity of the 3 euploid embryos obtained during the IVF cycle with the genomic DNA from the aneuploid POC

Sample ID	Similarity to POC gDNA
Embryo 3	74 %
Embryo 6	55 %
Embryo 7	75 %

To our knowledge this the only case of PGS and euploid SET resulted in a 45,X POC as consequence of a natural pregnancy. A similar event has been reported in a large study about twin births after SET where the authors demonstrated that about 1 in 5 twins is due to a concurrent spontaneous conception [4]. Another report described a case of dizygotic twins of different-sex delivered after SET demonstrated to be the consequence of a natural fertilization [2].

## Conclusions

Our case report demonstrates the occurrence of an aneuploid spontaneous conception, resulted in a miscarriage, in the course of a natural SET cycle. This case highlights the importance of avoiding intercourses and natural transfer cycles for couples with reproductive genetic risk, such as those undergoing preimplantation genetic diagnosis (PGD)/PGS cycles, in order not only to reduce the incidence of multiple pregnancies but especially to avoid the risk of aneuploid natural conceptions.

Moreover, cytogenetic analysis of the POCs and fingerprinting of the children born after PGS are strongly recommended.

Furthermore, with this study we confirm that parental DNA information is not necessary since discrimination was achieved based on a clear genotypic heterogeneity among samples, without incorporating selection of informative SNPs from parental genotype. Therefore, the additional time and expense of evaluating parental DNA could be avoided with this methodology as also previously reported [15].

## Abbreviations

a-CGH: Array-comparative genomic hybridization
AMH: Anti-Müllerian hormone
ART: Assisted reproductive technology
FISH: Fluorescence in situ hybridization
FSH: Follicle-stimulating hormone
GnRH: Gonadotropin-releasing hormone
ICSI: Intracytoplasmic sperm injection
IUI: Intrauterine insemination
IVF: In vitro fertilization
LH: Luteinizing hormone
PGD: Preimplantation genetic diagnosis
PGS: Preimplantation genetic screening
POC: Product of conception
qPCR: Quantitative polymerase chain reaction
rFSH: Recombinant FSH
rhCG: Recombinant human chorionic gonadotropin
SET: Single embryo transfer
SNPs: Single nucleotide polymorphisms
TE: Trophectoderm
